# Supplementary material for: The neglected role of abandoned cropland in supporting both food security and climate change mitigation
Source: Nat Commun. 2023 Sep 28;14:6083. doi: 10.1038/s41467-023-41837-y (PMC10539403; doi:10.1038/s41467-023-41837-y)
Supplement: Supplementary file 1 — Supplementary Information [file 41467_2023_41837_MOESM1_ESM.pdf]

Supplementary Information

TITLE

The neglected role of abandoned cropland in supporting both food security and climate change mitigation

AUTHORS

Qiming Zheng[1,2,*], Tim Ha[2], Alexander V. Prishchepov[3,4], Yiwen Zeng[2,5], He Yin[6], Lian Pin Koh[2,*]

1. Department of Land Surveying and Geo-Informatics, Hong Kong Polytechnic University, Hung Hom, Kowloon, Hong Kong
2. Centre for Nature-based Climate Solutions, National University of Singapore, Singapore 117546, Singapore
3. Department of Geosciences and Natural Resource Management (IGN), University of Copenhagen, Øster Voldgade 10, DK-1350 København K, Denmark
4. Center for International Development and Environmental Research (ZEU), Justus Liebig University, Senckenbergstraße 3, 35390 Giessen, Germany
5. School of Public and International Affairs, Princeton University, Princeton, NJ 08544, USA
6. Department of Geography, Kent State University, Kent, OH 44242, USA

[*] Corresponding authors:

Qiming Zheng (qiming.zheng@polyu.edu.hk)

Lian Pin Koh (lianpinkoh@nus.edu.sg)

CONTENTS

Supplementary Figures

1. Supplementary Fig. 1 | Abandoned cropland extent, climate change mitigation and food production potential at the continental scale.
2. Supplementary Fig. 2 | Possible outcomes of all simulated scenarios.
3. Supplementary Fig. 3 | Integrated cropland productivity from 15 selected crops and carbon sequestration rates.
4. Supplementary Fig. 4 | Priority for recultivating and reforesting abandoned cropland for representative scenarios.
5. Supplementary Fig. 5 | Recultivated abandoned cropland.
6. Supplementary Fig. 6 | The spatial allocation map of 15 selected crops.
7. Supplementary Fig. 7 | Breakdowns of the climate change mitigation potential.
8. Supplementary Fig. 8 | Area with production gap under rain-fed condition.
9. Supplementary Fig. 9 | Area with production gap under irrigated condition.
10. Supplementary Fig. 10 | Recultivation priorities when different yield improvement approaches are applied.
11. Supplementary Fig. 11 | Percentage of protected area.
12. Supplementary Fig. 12 | Reforestation costs at a global scale (a) and in abandoned cropland suitable for reforestation (b). Carbon sequestration rates at abandoned cropland suitable for reforestation (c).
13. Supplementary Fig. 13 | Spatial distribution of validation samples.
14. Supplementary Fig. 14 | Recultivatable area and food production potential of different threshold settings.
15. Supplementary Fig. 15 | Relationship between percentage of selected input data and the resulting extent of recultivatable abandoned cropland.
16. Supplementary Fig. 16 | Sensitivity analysis on the achievable climate change mitigation potential.

Supplementary Texts

1. Supplementary Text 1| Additional population that can be fed by the food production potential from recultivating abandoned cropland.
2. Supplementary Text 2 | Breakdown of net climate change mitigation potential.
3. Supplementary Text 3 | Approaches to increase achievable food production potential and climate change mitigation potential.
4. Supplementary Text 4 | Impact of local land-use regulation on the availability of abandoned cropland.
5. Supplementary Text 5 | Challenges in incorporating socioeconomic factors into spatially-explicit trade-off analysis.
6. Supplementary Text 6 | Accuracy assessment of our identified abandoned cropland.
7. Supplementary Text 7 | Factors used for modelling suitability of recultivation.
8. Supplementary Text 8 | Sensitivity analysis of recultivatability threshold.
9. Supplementary Text 9 | Sensitivity analysis of achievable climate change

mitigation potential.

10. Supplementary Text 10 | Scenario simulation process.

References

SUPPLEMENTARY FIGURES

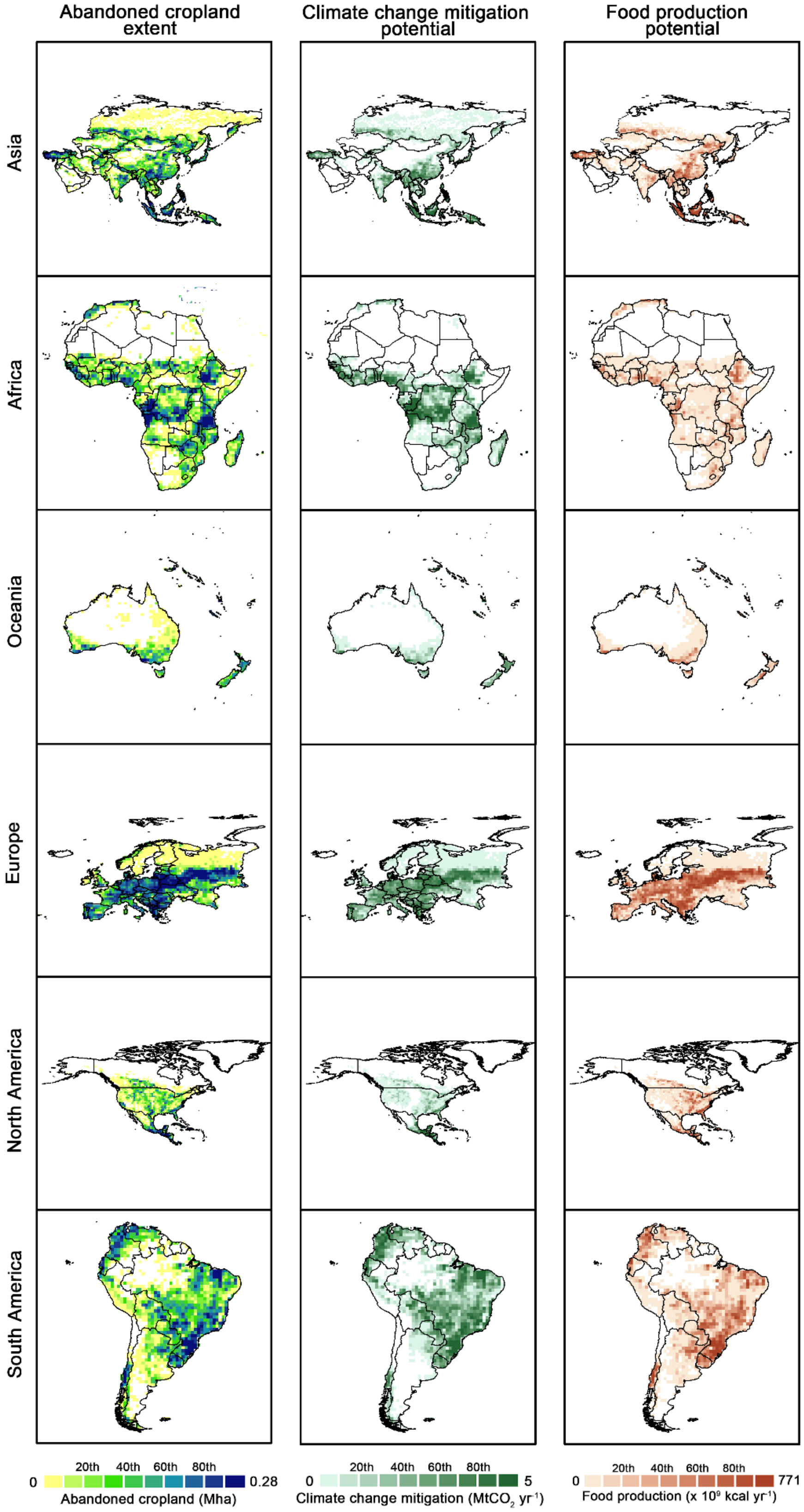

Supplementary Fig. 1 | Abandoned cropland extent, maximum net climate change mitigation potential, and maximum food production potential breakdown for each continent.

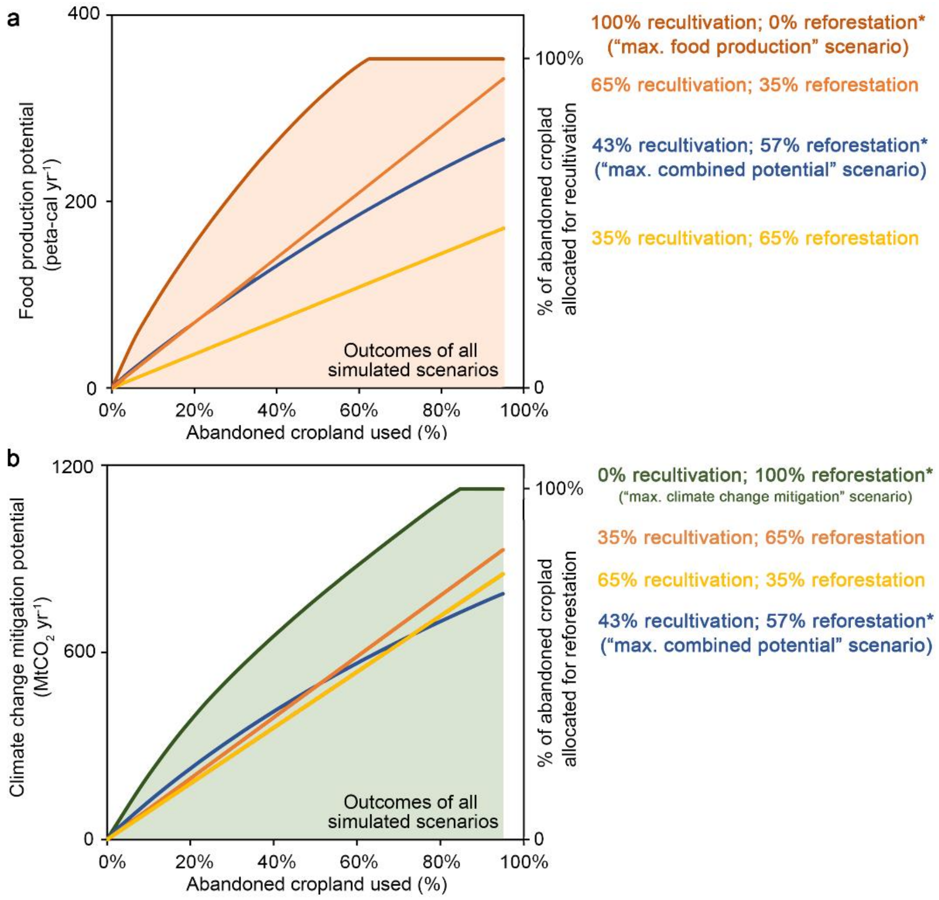

Supplementary Fig. 2 | Possible outcomes of all simulated scenarios, where * indicates the scenario employing spatial prioritization. The lines show eight examples of simulated outcomes. The right y-axis, as well as the values in the text of eight examples, show the percentage of abandoned cropland to be allocated for recultivation and reforestation out of the total abandoned cropland to be used.

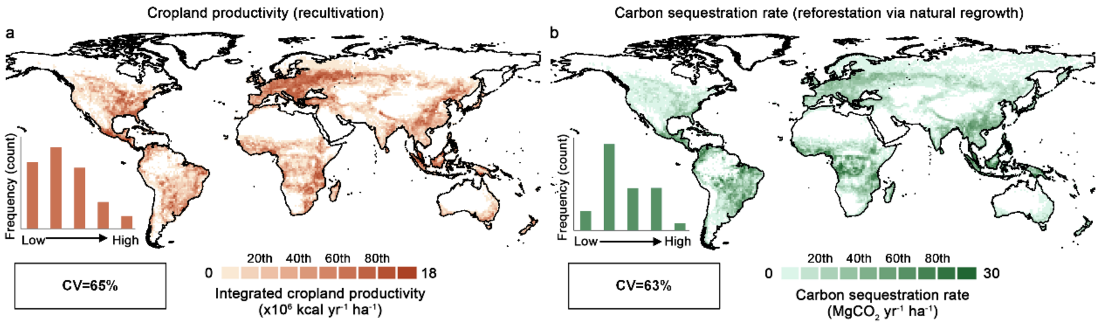

Supplementary Fig. 3 | Integrated cropland productivity from 15 selected crops (a) and carbon sequestration rates (b). The histograms indicate the frequency distribution of integrated cropland productivity and carbon sequestration rates in global abandoned cropland. Coefficient of variation (CV) of cropland productivity and carbon sequestration rates of abandoned cropland is also provided.

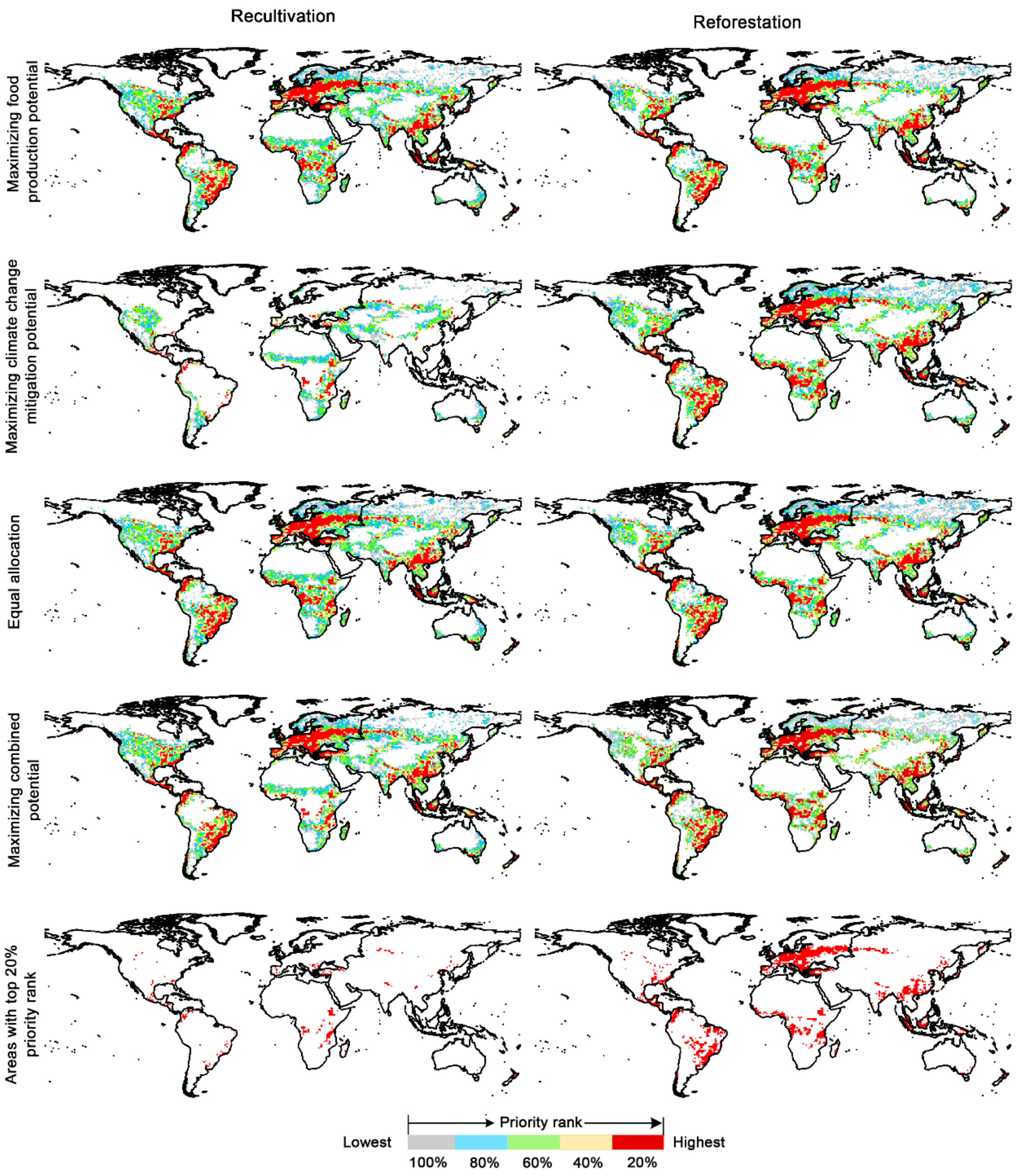

Supplementary Fig. 4 | Priority of recultivating and reforesting abandoned cropland for four representative scenarios and areas with the top 20% priority rank. Priority ranks are determined by the integrated crop productivity and carbon sequestration rate of pixel each pixel under each scenario.

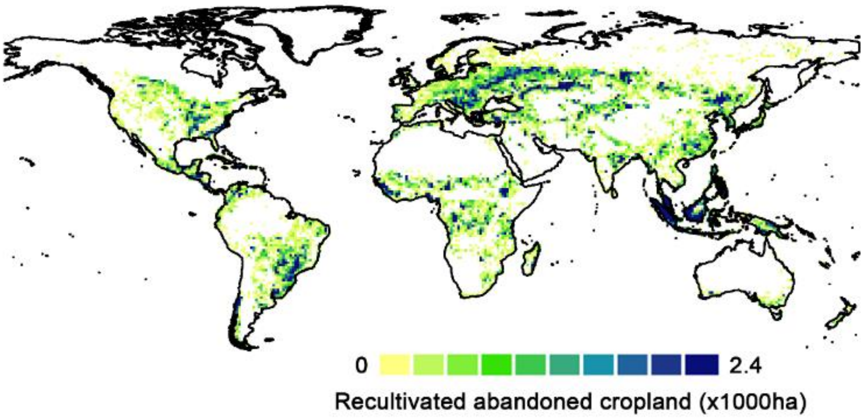

Supplementary Fig. 5 | Recultivated abandoned cropland during 1992-2020 (x1000 ha). Each pixel is 1 arc-degree resolution aggregated from 10 arc-second.

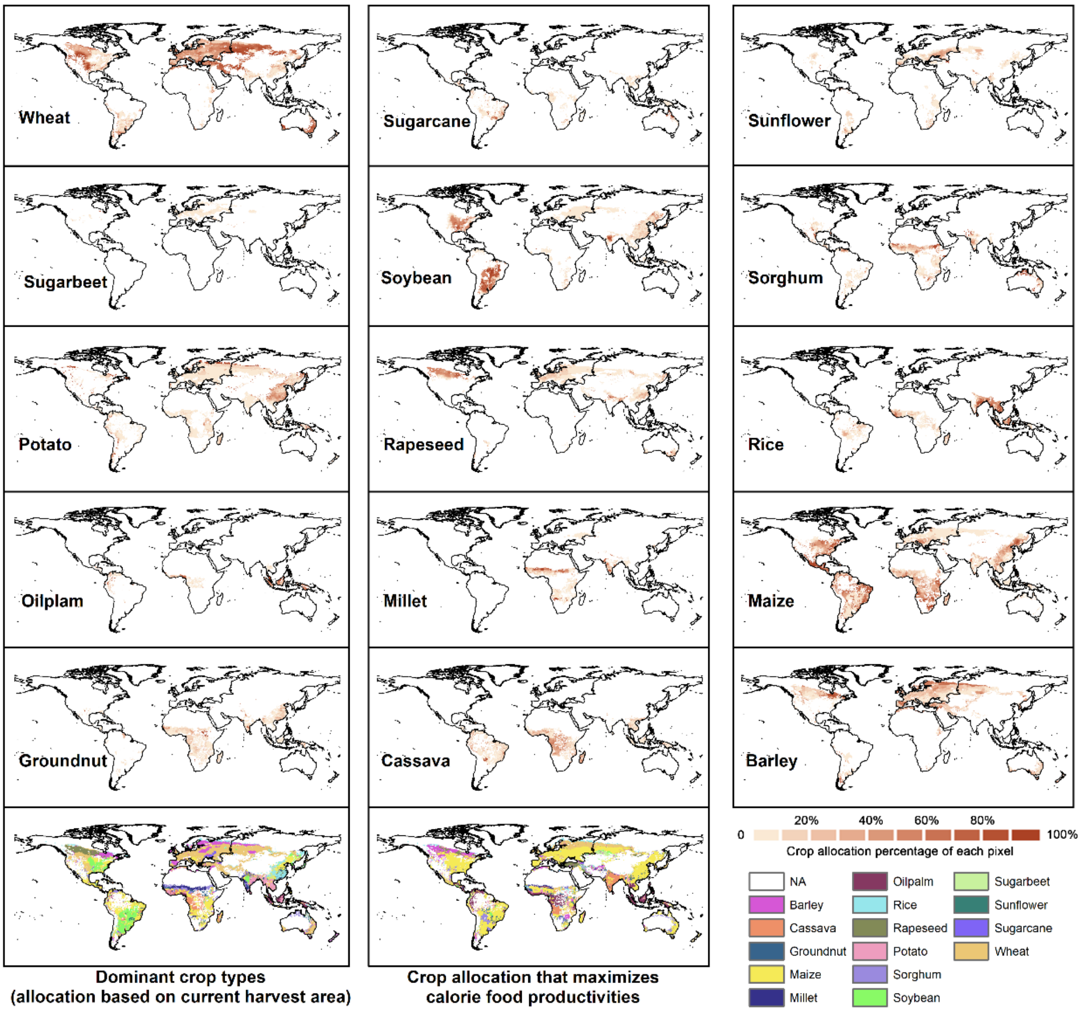

Supplementary Fig. 6 | The spatial allocation map of 15 selected crops (%) and the corresponding dominant crop types of each 5arcmin pixel (under rain-fed and present-day actual yield condition). The crop allocation is based on the current harvest area of each crop. The crop allocation that maximizes calorie food productivity is also provided.

SUPPLEMENTARY TEXTS

Supplementary Text 1 | Additional population that can be fed by the food production potential from recultivating abandoned cropland

In this study, if all abandoned cropland suitable for crop production is recultivated, it can increase the food supply by 363 Peta-calories yr^{-1}. We calculated how many people this amount of food could feed based on three different diets and calorie intake conditions, including a healthy diet (2085 kcal day^{-1} $person^{-1}$; BMI of 22.5), current average calorie intake condition (2890 kcal day^{-1} $person^{-1}$) and calorie intake condition in 2050 (3320 kcal day^{-1} $person^{-1}$) based on Clark, et al. [1]. Our results show that around 292 million people (calorie intake condition in 2050), 345 million people (current calorie intake condition), and 476 million people (healthy diet) could be fed.

Supplementary Text 2 | Breakdowns of net climate change mitigation potential

In our study, net climate change mitigation potential in the next 30 years by considering the carbon sequestration achievable from allowing forest natural regrowth on abandoned cropland and the emissions from clearing historically accumulated aboveground biomass carbon for recultivation. In addition to the spatial pattern and total amount of climate change mitigation potential shown in the main text, here we present the breakdowns of mitigation potential, including carbon sequestration obtained from reforestation via natural regrowth and emissions from clearing the historically accumulated biomass carbon for recultivation (without energy recovery) (**Supplementary Fig. 7**).

We also present the lost mitigation potential of foregone natural regrowth if the abandoned cropland suitable for natural regrowth and recultivation is recultivated. This helps to reveal the opportunity "cost" of recultivation. It should be noted that the lost mitigation potential of foregone natural regrowth is not included in the net climate change migration potential calculation because the one-time emission from land clearing has been accounted for.

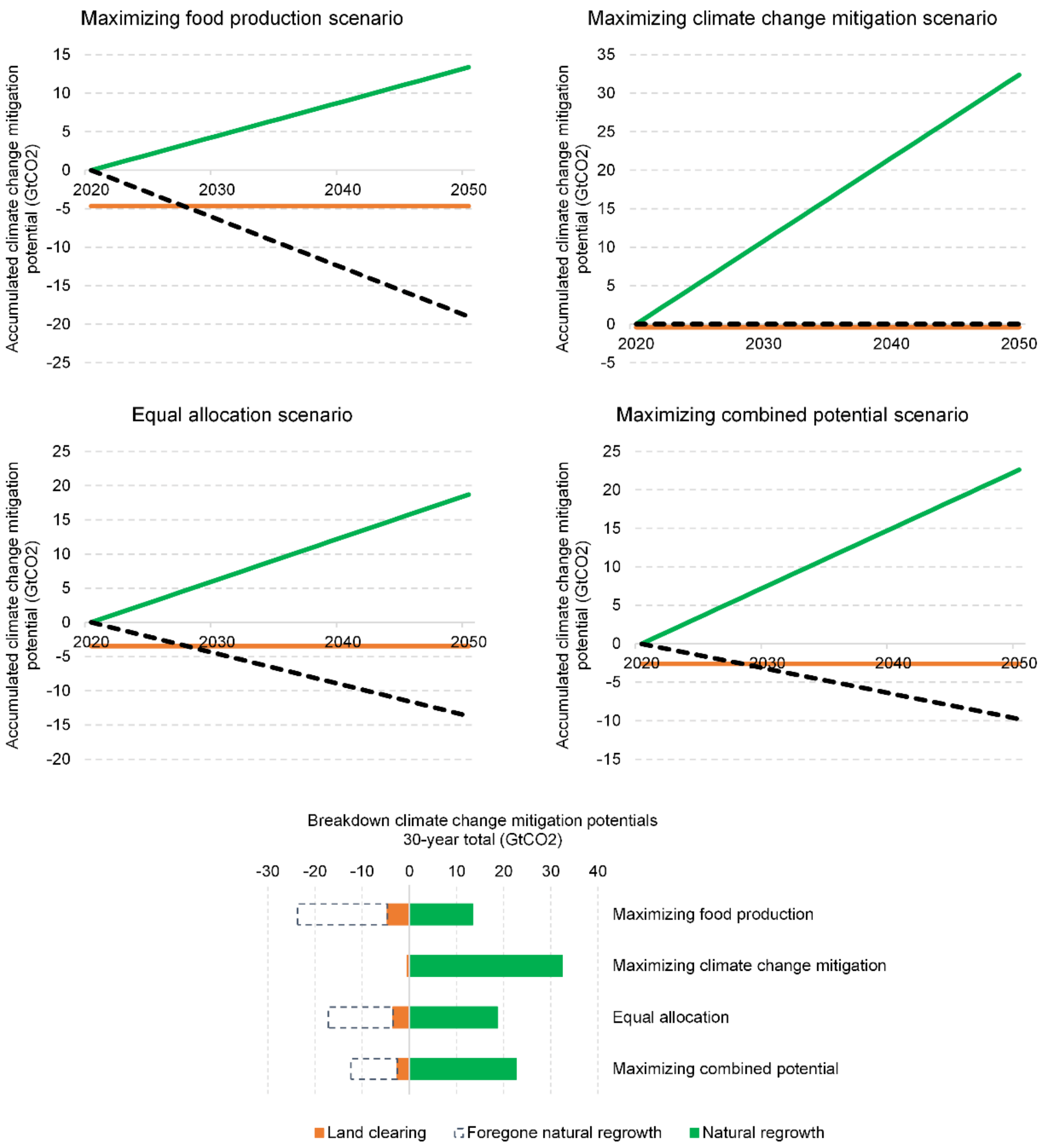

Supplementary Fig. 7 | Breakdowns of the climate change mitigation potential, including carbon sequestration obtained from reforestation via natural regrowth, emissions from clearing the historically accumulated biomass carbon for recultivation (without energy recovery), and the lost mitigation potential of foregone natural regrowth if the abandoned cropland is recultivated.

Supplementary Text 3 | Approaches to increase achievable food production potential and climate change mitigation potential

Our estimates of the food production potential and climate change mitigation potential are conservative. There are a few possible approaches to improve the achievable potentials and free up land for other use. We considered the following possible approaches and quantitatively assessed their benefits in improving the achievable potential of abandoned croplands.

(1) Food production potential

Halving food waste and loss

When modelling food production potential, about 17-34% of food produced was considered either wasted or lost during production, supply chain, and consumption. We estimated the food production potential, if global food waste and loss can be halved, as target 12.3 of the SDGs envisions[1]. See country-specific food waste and loss statistics in **Supplementary Data 3**.

Improving water supply

Our original estimate of crop productivity was based on yields under a rain-fed conditions. We estimated the food production potential if irrigation becomes available.

Data source: GAEZ v4 database[2] (15 selected crops, actual yield, 2010, irrigated; **Supplementary Table 1**).

Improving crop yields

Our original estimate of crop productivities was based on the present-day actual yield of each selected cropland of GAEZ database, which was downscaled and averaged from national statistics of 2009-2011 (FAOSTAT). We used this actual yield to conservatively estimate the food production potential for 2020-2050, reflecting the less favorable biophysical, socioeconomic and climatic conditions in abandoned cropland. We estimated the food production potential if the crop yields can be improved from present-day actual yield to 30-year mean attainable yields in 2010-2040 with high agriculture input, as a consequence of agricultural intensification and agricultural technology advancements. High agricultural input reflects a modern agriculture condition, assuming that "the farming system is mainly market-oriented and production is based on improved or high yielding varieties that are fully mechanized where possible with low labor intensity and uses optimum applications of nutrients and chemical pest, disease and weed control" (Definition provided in GAEZ document). The attainable yields are the ensemble mean of five climate models GFDL-ESM2M, HadGEM2-ES, IPSL-CM5A-LR, MIROC-ESM-CHEM, and NorESM1-M, obtained from the bias-corrected Intersectoral Impact Model Intercomparison Project (ISI-MIP)[3]. We considered attainable yields under different water supply (rain-fed/irrigated) and climate forcing (RCP4.5/RCP8.5). We only applied such yield improvements in suitable abandoned cropland with identified yield gap (**Supplementary Figs. 8&9**).

Data source: GAEZ v4 database[2] (15 selected crops, attainable yield, 2010-2040, rain-fed/irrigated, high inputs, ensemble climate data, RCP4.5/RCP8.5, with CO2 fertilization)

Optimizing crop allocation

The crop allocation of each 5arcmin pixel was based on the area-weighted present-day harvest area of each crop. However, if we optimal the crop allocation by selecting the

crop with the highest yield to maximize the crop productivity of each pixel, we found that the food production potential could be increased by 74% (**Supplementary Table 2; Supplementary Fig. 6**). It should be noted that because such optimized crop allocation is highly hypothetical, so we did not present this results in the main text.

Data source: GAEZ v4 database[2] (15 selected crops, actual yield, 2010, rain-fed; 15 selected crops, harvest area, 2010)

Impacts of future climate change

To present the impact of future climate change on crop yields and food production potential on abandoned cropland, we compared food production potential with attainable yields during 2010-2040 and 2040-2070, under different water supply (rain-fed/irrigated) and climate forcing (RCP4.5/RCP8.5) (**Supplementary Table 3**).

Data source: GAEZ v4 database[2] (15 selected crops, attainable yield, 2010-2040/2040-2070, rain-fed/irrigated, high inputs, ensemble climate data, RCP4.5/RCP8.5, with CO2 fertilization)

Analysis

We further analyzed (1) how an increase in crop yields would affect spatial prioritization of recultivation (**Supplementary Fig. 10**) and (2) how an increase in crop yields would free abandoned cropland for reforestation if we maintain the food production at our original estimation (**Fig. 4**).

Discussion 1 – Impact of future climate change on crop yields

It should be noted that due to data availability, the future crop yield data are attainable yields, which reflect crop yields under optimal management conditions (e.g., i.e., recommended plant density, non-limiting nutrient condition, effective control of biotic stresses, etc.)[4]. The actual future crop yields might be subject to many environmental constraints (e.g., nitrogen stress). Second, while our analysis showed a marginal increase in future crop yields in 2010-2040 and 2040-2070, we highlight that (1) the responses of crop yields to future climate warming differ greatly across space (latitude) and crop types. As reported in ref.[5], in general, mid- and high-latitude areas with moderate temperature increase (1-3°C) and the associated CO2 and rainfall changes would lead to crop yield increase. In contrast, crop yields in low-latitude areas would be subject to the negative impact of climate warming. (2) Our study focused on mid-term projections of future crop yields, i.e., 2010-2040 and 2040-2070. However, the end of the century will see a higher temperature increase (RCP4.5: 1.1 to 2.6°C; RCP8.5: 2.6 to 4.8°C)[a]. Such substantial warming is estimated to lead to a prevailing decrease in crop yields[6].

[a] IPCC AR5: https://web.archive.org/web/20181223030119/https://archive.ipcc.ch/report/ar5/wg1/

Discussion 2 – GAEZ crop yields validation

GAEZ model and its outputs have been validated by (1) comparisons against other model outputs[5,7], (2) comparisons against statistical records[8], and (3) comparisons against crop yields map estimated by bottom-up approaches (i.e., models based on local observations), such as GYGA data[9,10]

Nevertheless, it should be noted that the above-mentioned investigations are more or less limited in multiple aspects: (1) crop types: Grogan, et al. [7] validated the crop yields of 4 crops and harvest areas of 25 crops and Rosenzweig, et al. [5] validated the crop yields of 4 crops; (2) GAEZ model version: Rattalino Edreira, et al. [9] is based on crop yields of GAEZ v3 product, while Rosenzweig, et al. [5] is based on GAEZ-IMAGE product; and (3) study areas: Grogan, et al. [7] validated the global-scale crop yields against Global Dataset of Historical Yields (GDHY) product, while Deng, et al. [10] only validated crop yields for China.

Despite these limitations in GAEZ v4 product validation, GAEZ v4 has been one of the most widely-used datasets for providing global-scale estimates of including harvest areas and crop yields[11,12]. Future validation efforts that are at global scale and cover more crop types are of crucial necessity.

(2) Climate change mitigation potential

Actively reforest suitable abandoned cropland outside global protected areas

When modelling climate change mitigation, we considered the carbon sequestration rates based on natural forest regeneration to avoid adverse impacts on local biodiversity[13]. However, the carbon sequestration rates of natural forest regeneration are particularly lower in non-tropical areas than reforestation approaches with human interventions (e.g., active reforestation or afforestation). The accumulated aboveground biomass carbon via natural regeneration is reported to take more than 60-80 years to recover to 90% of the old-growth forest level[14,15]. Also, a recent study indicates that abandoned cropland under the natural regeneration process would face intense threats of being recultivated unless additional policy interventions are implemented[16].

We estimated the increase in achievable climate change mitigation potential if we actively reforest abandoned cropland (68 Mha, 82%) outside global protected areas defined by IUCN and UNEP (**Supplementary Fig. 11**). We assumed a linear relationship between the carbon sequestration rates of natural regeneration and active reforestation, with a conversion factor of 1.71±0.58 (mean±std, n=27) based on our literature review (**Supplementary Data 4**). We applied this conversion factor to

estimate the climate change mitigation potential of actively reforesting abandoned cropland outside global protected areas, together with reforesting abandoned cropland via natural regrowth inside global protected areas.

We acknowledge this was a simplified estimation because the carbon sequestration rates may follow non-linear relationship[17,18] and we did not account for the variations in terms of geographical regions, climate zones, biomes, or plant functional types. Nevertheless, it should be noted that the purpose of this analysis is to provide readers with a rough and supplementary understanding of how much additional climate change mitigation potential we can achieve on abandoned cropland. Besides, global average carbon sequestration rates via active reforestation estimated in our study (5.6 MgC yr^{-1} ha^{-1}) is consistent with estimation from another study (e.g., 6.0 MgC yr^{-1} ha^{-1})[19].

Analysis

We analyzed how the additional climate change mitigation made available by active reforestation would free up suitable abandoned cropland for recultivation, assuming we maintain climate change potential at our original estimates.

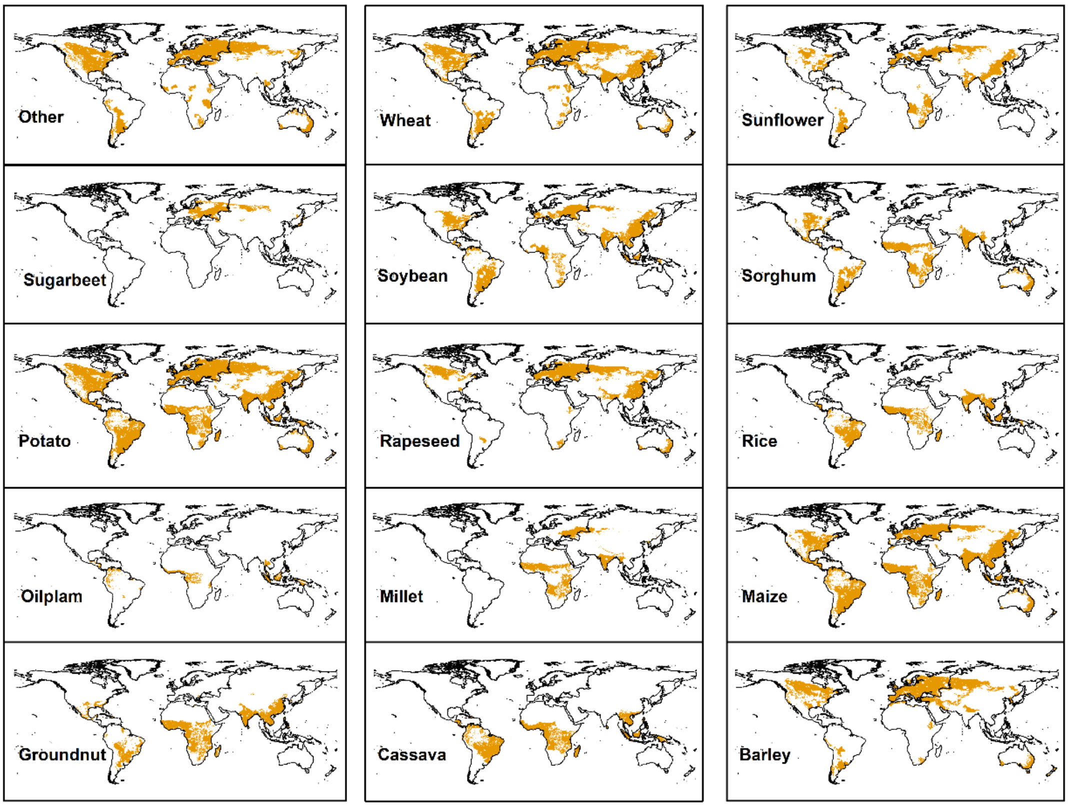

Supplementary Fig. 8 | Area with production gap under rain-fed conditions.

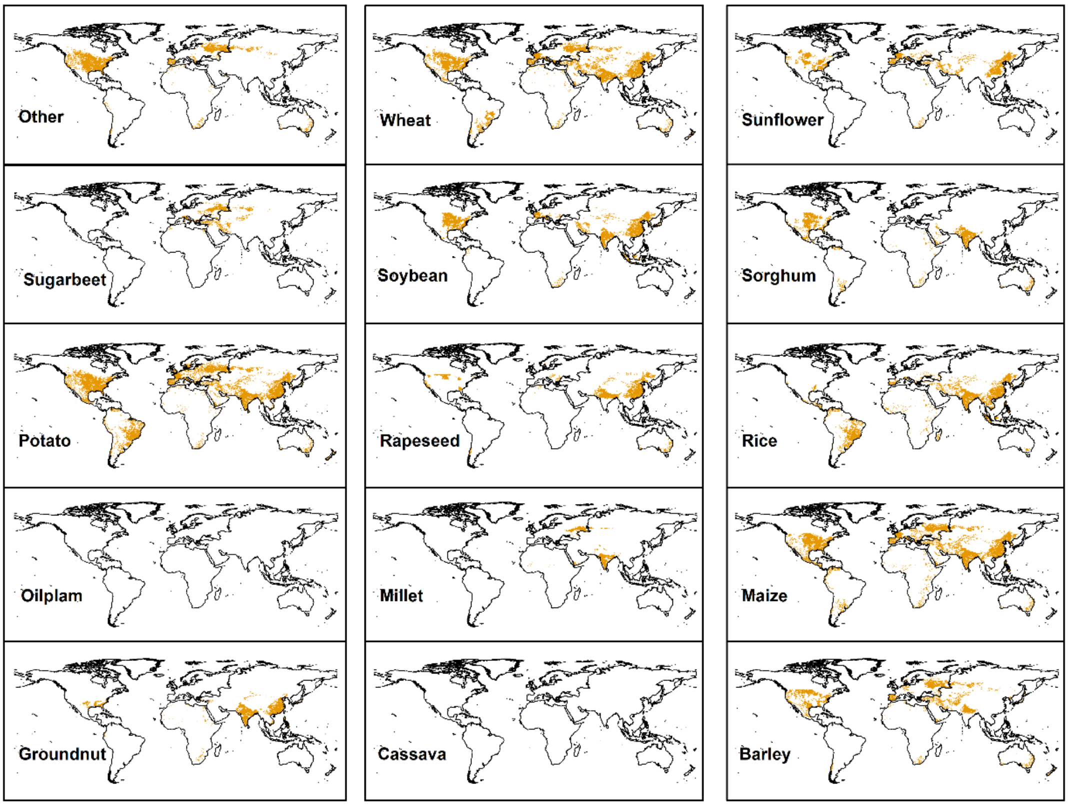

Supplementary Fig. 9 | Area with production gap under irrigated conditions.

Supplementary Table 1 | Yield data used in this study and the associated settings. *: Only yields with high agricultural input (high-level inputs) are available in the GAEZ database. High-level inputs refer to the condition that the farming system is mainly market-oriented with advanced agricultural management. Production is based on improved or high-yielding varieties, is fully mechanized where possible with low labor intensity and uses optimum applications of nutrients and chemical pest, disease and weed control[2].

**Yield type**	**Period**	**Water supply**	**Input level**	**Climate data source**	**Climate forcing**
Actual yield (Main analysis)	2010	Rainfed Irrigated	NA	CRUTS-32	Historical
Attainable yield (Supplementary analysis)	2010-2040	Rainfed Irrigated	High*	Ensemble (Ensemble mean of all models' outputs)	RCP 4.5 RCP 8.5
Attainable yield (Supplementary analysis)	2040-2070	Rainfed Irrigated	High*	Ensemble (Ensemble mean of all models' outputs)	RCP 4.5 RCP 8.5

Supplementary Table 2 | The amount of abandoned cropland allocated for recultivation and reforestation and the corresponding food production potential and climate change mitigation potential when the crop is in an optimal allocation that maximizes calorie food productivities (see Methods for detailed scenario narratives). *:

Values in parentheses specify the percentage of abandoned cropland allocated, for either recultivation or reforestation, out of the total suitable extent for recultivation (61 Mha, 100%) and reforestation (83 Mha, 100%) or the percentage of the achieved potential out of the maximum achievable food production potential (635 Pcal yr^{-1}, 100%) and climate change mitigation potential (1,080 $MtCO_2$ yr^{-1}, 100%). **: The combined potential (A+B; %) is calculated by the sum of achieved food production potential (**A**; %) and climate change mitigation potential (**B**; %), which serves as a hypothetical indicator to evaluate the integrated outcome of each scenario.

**Representative scenarios**	**Abandoned cropland for recultivation Mha (% of max.)***	**Abandoned cropland for reforestation Mha (% of max.)**	**Food production potential Pcal yr^{-1} (% of max.; A)**	**Net climate change mitigation potential $MtCO_2$ yr^{-1} (% of max.; B)**	**Combined potential (A+B)****
Maximizing food production	61 (100%)	33 (39%)	635 (100%)	290 (27%)	127%
Maximizing climate change mitigation	11 (18%)	83 (100%)	56 (9%)	1,066 (99%)	108%
Equal Allocation	47 (77%)	47 (56%)	473 (74%)	508 (47%)	121%
Maximizing combined potential	43 (70%)	51 (61%)	513 (81%)	653 (60%)	141%

Supplementary Table 3 | Impact of future climate change on crop yields and achievable food production potential on current abandoned cropland (original estimate based on rain-fed actual yield of 2010: 363 Pcal yr^{-1}).

**Climate forcing**	**Water supply**	**2010-2040**	**2040-2070**	**Diff.**
RCP4.5	Rain-fed	506 Pcal yr^{-1}	522 Pcal yr^{-1}	+3%
	Irrigated	728 Pcal yr^{-1}	814 Pcal yr^{-1}	+12%
RCP8.5	Rain-fed	508 Pcal yr^{-1}	523 Pcal yr^{-1}	+3%
	Irrigated	749 Pcal yr^{-1}	823 Pcal yr^{-1}	+10%

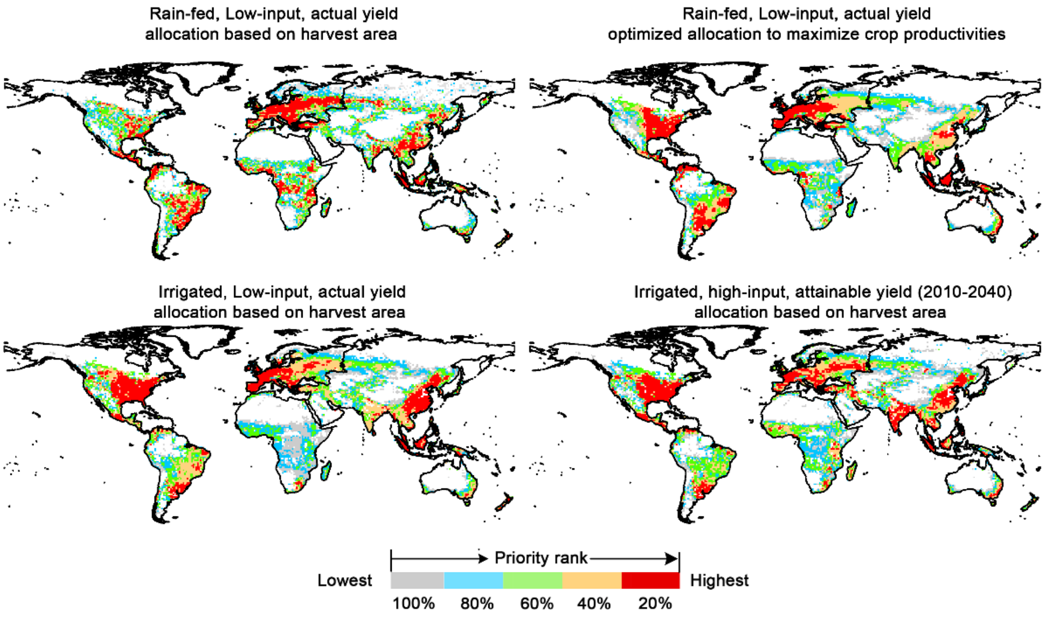

Supplementary Fig. 10 | Recultivation priorities when different yield improvement approaches are applied.

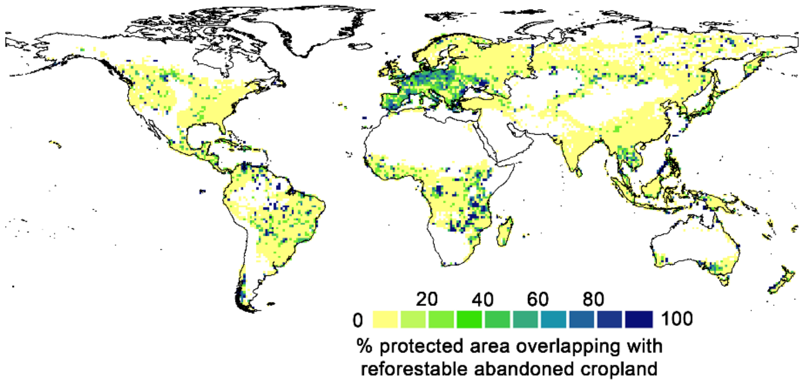

Supplementary Fig. 11 | Percentage of protected area overlapping with reforestable abandoned cropland in each 1 deg pixel. Number of reforestable abandoned cropalnd pixels in projected areas / Number of reforestable abandoned cropland pixels.

Supplementary Text 4 | Impact of local land-use regulation on the availability of abandoned cropland

One of the societal issues that we did not consider is the impact of local land-use planning. We exemplify this impact by oil palm plantations in the tropics. If abandoned cropland is assigned for palm oil production (a common phenomenon in tropical countries where concessions are granted by local government or other legal entities), this area of abandoned cropland would then be unavailable for recultivation and reforestation.

To determine how such an issue would affect the availability of abandoned cropland, we overlaid our identified abandoned cropland with the latest map of global oil palm planations[20]. We found that about 0.66 Mha of abandoned cropland would become unavailable for recultivation and reforestation due to spatial overlapping with oil palm plantations.

Supplementary Text 5 | Challenges in incorporating socioeconomic factors into spatially-explicit trade-off analysis

In this study, we only considered the trade-offs between recultivation and reforestation in terms of their suitability and potential but not other socioeconomic factors at a context-specific scale, including costs of recultivation and reforestation. This is mainly because of the lack of global scale maps explicitly presenting the spatial variation of these factors. Taking the costs of recultivation and reforestation as examples, existing estimates and mappings of global recultivation and reforestation costs can only present spatial variation at the country level, where pixels within a country exhibit the same or similar cost values. In contrast, our food production potential and climate change mitigation potential maps show high spatial variation across space (Extended Data Fig. 3). This difference in the available level of spatial details makes it difficult to integrate costs into our trade-off assessment and spatial priority mapping.

Furthermore, we present an attempt to estimate global active reforestation costs. We compared the spatial variation between the carbon sequestration rates map used in our study and the reforestation cost map estimated by a widely-adopted approach of recent studies[21,22]. Specifically, following the approach of Strassburg, et al. [21], we used the average implementation costs for reforestation projects in Brazil (2,148 USD ha^{-1}), which was obtained from an early study for the Atlantic forest[23]. The reforestation costs in other countries were estimated based on their costs of agricultural labor and fertilizer input relative to Brazil's. The agricultural costs and fertilizer inputs were assumed to account for 70% and 30% of reforestation costs based on evidence from previous projects in Brazil[24].

We then compared the variation of the reforestation costs map and carbon sequestration rates map, measured by the coefficient of variation (CV, %), across three spatial scales: (1) within each country, (2) within the same economic region (five economic regions defined by Riahi, et al. [25]), and (3) across the globe (**Supplementary Fig. 12 and Supplementary Table 4**).

Supplementary Table 4 | Coefficient of variation (CV, %) across three spatial scales.

	**Within a country (mean±std)**	**Within a region (mean±std)**	**Across the globe**
**Reforestation cost**	0%	32±40%	59%
**Carbon sequestration rate**	29±18%	55±8%	65%

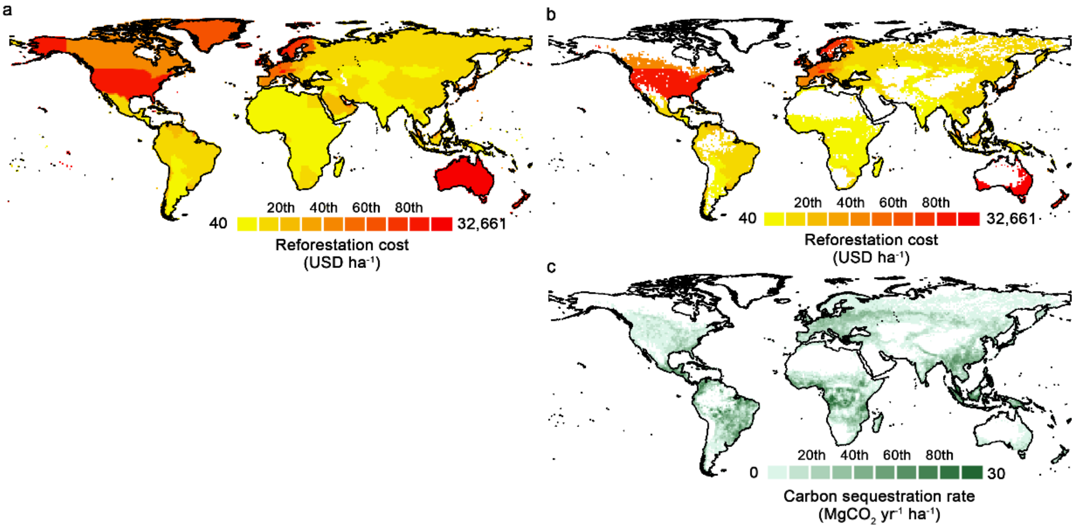

Supplementary Fig. 12 | Reforestation costs at a global scale (a) and in abandoned cropland suitable for reforestation (b). Carbon sequestration rates at abandoned cropland suitable for reforestation (c).

Our estimated carbon sequestration rates map illustrates the spatial heterogeneity within countries (CV=29±18%). In contrast, reforestation costs are constant for all pixels within each country (CV=0%). However, the reforestation costs are supposed to vary within each country, depending on travel distance, biophysical conditions (e.g., water supply and soil fertility), labor availability[26], etc. The variation is also better captured in the carbon sequestration rates map than the reforestation costs map at regional (55% vs. 32%) and global scales (65% vs. 59%).

Based on our analysis, we conclude that: (1) reforestation costs estimated by the commonly-adopted approach are more suitable to present variation, at least, across different countries or regions, but not within a country, although a high variation within the country is expected; (2) spatial variation is better illustrated in the carbon sequestration rates map than reforestation costs map; and (3) despite the necessity, incorporating reforestation costs and other socioeconomic factors into spatially-explicit trade-off analysis is still very challenging.

Supplementary Text 6 | Accuracy assessment of our identified abandoned cropland

In our study, we used two approaches to validate our abandoned cropland map. First, we followed Eq. 13 of Olofsson, et al. [27] to determine the initial sample size for abandoned cropland class (57 samples) and non-abandoned cropland class (828 samples). Then, to avoid problems with small sample sizes for rare thematic classes (abandoned cropland), we used disproportionate stratified sampling[28] by selecting 828 samples for both abandoned cropland class and non-abandoned cropland class, thus in total 1656 validation samples (**Supplementary Fig. 13**). Overall accuracy, producer's accuracy, user's accuracy, and F1 score to account for possible sampling bias[28]

(**Supplementary Table 5**). These accuracy scores are also provided on a continental basis (**Supplementary Table 6**). In addition, we calculated the 95% confidence intervals of the total abandoned cropland extent (101±35 Mha)[29].

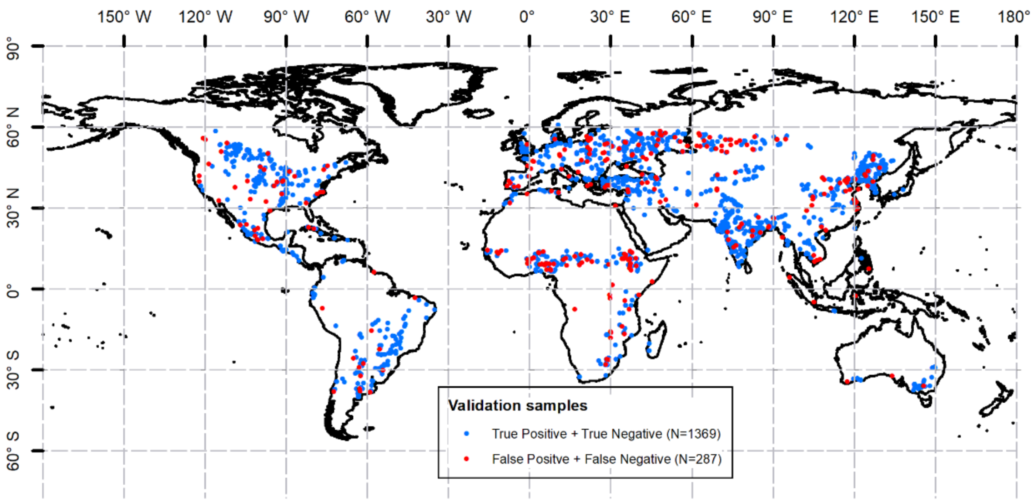

Supplementary Fig. 13 | Spatial distribution of validation samples.

Supplementary Table 5 | Confusion matrix at the global level.

	**Reference**	
	Abandoned cropland	Non-abandoned cropland
Abandoned cropland	773	55
Non-abandoned cropland	232	596
**Accuracy assessment indices**		
Overall accuracy	0.828	
F1 score	0.843	
Producer's accuracy of abandoned cropland	0.770	
User's accuracy of abandoned cropland	0.934	
Abandoned cropland extent error (95% CI)[29]	±35 Mha	

Supplementary Table 6 | Confusion matrix for each continent. *: Not available due to a limited number of validation samples.1

**Continents**	**Overall accuracy**	**User's accuracy**	**Producer's accuracy**	**F1 score**
Africa	0.76	0.86	0.71	0.77
Asia	0.84	0.96	0.79	0.87
Australia	0.8	NA*	0.63	NA*
Europe	0.79	0.92	0.75	0.83
North America	0.85	0.95	0.79	0.86

South America	0.88	0.84	0.80	0.82

Second, we also qualitatively compared the abandoned cropland identified in our study with previous studies (n=42; **Supplementary Data 1**). Note that periods, resolutions, land classification methods, differences in land-use class definition, and abandonment criteria vary between studies, so a quantitative comparison is not practical. Our abandoned cropland map is well consistent with existing studies in terms of abandonment extent and spatial pattern, particularly for those relying on the same input dataset (i.e., ESA-CCI).

Supplementary Text 7 | Driving factors for modelling suitability of recultivation

The driving factors that we considered to model recultivation suitability are listed in **Supplementary Table 7**.

Supplementary Table 7 | Factors used for modelling suitability of recultivation.

Variables	Source
Agro-ecological suitability	Fischer, et al. [2]
Market accessibility	Verburg, et al. [30]
Travel time to settlement	Nelson, et al. [31]
Population density	https://landscan.ornl.gov/
Cropland density	Fraction of cropland (based on ESA-CCI cropland in 2020) within each 9km x 9km neighbor.
Distance to stable cropland	Distance between a recultivated abandoned cropland pixel to the most adjacent stable cropland pixel. Stable cropland pixel is defined as a pixel that remains cropland during our study period (1992-2020).
Abandoned cropland density	Fraction of abandoned cropland identified in this study within each 9km x 9km neighbor.
INFORM Risk Index	A risk assessment index for natural and human-induced disasters produced by European Commission[32]
Average annual economic loss due to major natural hazard	GAR Risk Atlas of the United Nations Office for Disaster Risk Reduction[33]

Supplementary Text 8 | Sensitivity analysis of recultivation suitability modelling

In our study, we used historically recultivated abandoned cropland (1992-2020) and driving factors of recultivation to train and tune a MaxEnt model, and then estimated abandoned cropland prone to recultivation (recultivatability$\in$[0,1]) (see Methods). The final model reached an acceptable accuracy of AUC=0.79. In our analysis, we set a threshold of recultivatability >0.2 to identify recultivatable abandoned cropland, while

the remaining abandoned cropland was considered unlikely to be suitable for recultivation owing to substantial biophysical, ecological, and socioeconomic constraints. Such threshold setting resulted in 60% (61 Mha) of identified abandoned cropland being recultivatable and a maximum achievable food production potential of 363 Pcal yr^{-1}.

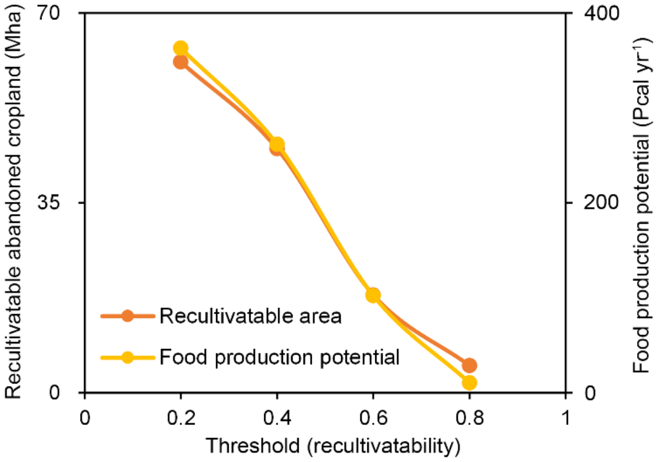

Supplementary Fig. 14 | Recultivatable area and food production potential of different threshold settings (a).

Here, we present the outcomes of different threshold settings (**Supplementary Fig. 14**). The recultivatable area and food production potential decrease from 61 Mha to 5 Mha and from 363 Pcal yr^{-1} to 7 Pcal yr^{-1}, respectively, when the threshold is changed from >0.2 to >0.8. Setting the threshold of recultivatability>0.2 is in line with how GAEZ database sets agro-ecological suitability (i.e., suitability index >0.2 implies at least marginal suitability for production[24]).

In addition, our original model used 2.8 Mha (about 300,000 pixels) of historically recultivated abandoned cropland as input data to train MaxEnt model and map abandoned cropland that is suitable for recultivation (**Supplementary Fig. 5**). We designed a sensitivity analysis to reflect how the input historical recultivation map would, in turn, affect the modelled suitability of recultivation. We randomly selected 95%, 90%, ... 10% of historically recultivated abandoned cropland as input training data and analyzed how the resulting extent of abandoned cropland that is suitable for recultivation would be affected.

Our analysis indicates that as long as there are a sufficient number of input data (historical recultivated abandoned cropland pixels), resulting extent of abandoned cropland suitable for recultivation is relatively stable (**Supplementary Fig. 15**).

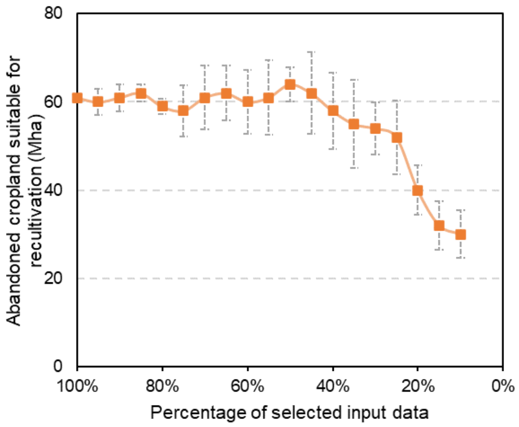

Supplementary Fig. 15 | Relationship between the percentage of selected input data (historically recultivated abandoned cropland) and the resulting extent of recultivatable abandoned cropland.

Supplementary Text 9 | Sensitivity analysis of achievable climate change mitigation potential

We evaluated the uncertainty of our estimated climate change mitigation potential with the corresponding pixel-by-pixel error rate provided by Cook-Patton, et al. [34]. The error rate is presented as the standard deviation of 100 random forest models' predictions. We simulated the outcomes with mean±std under spatially prioritized allocation scenarios and randomized allocation scenarios (**Supplementary Fig. 16**).

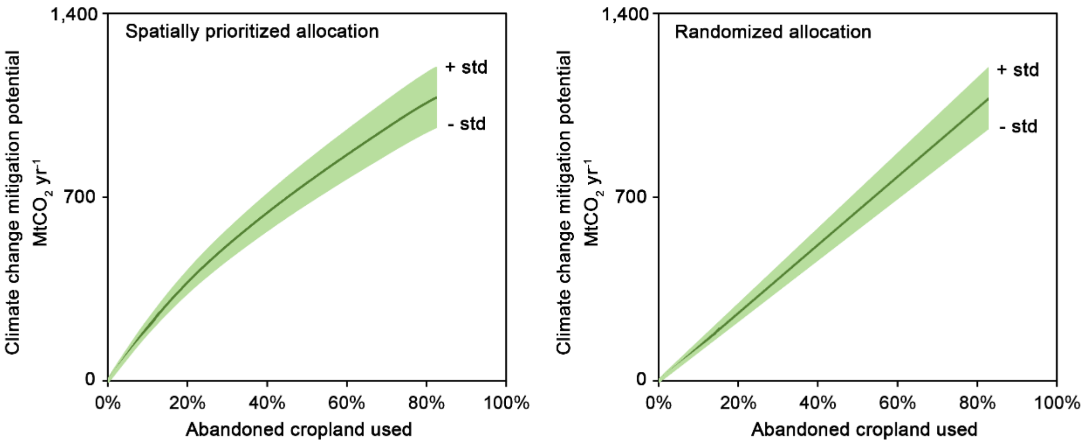

Supplementary Fig. 16 | Sensitivity analysis on the achievable climate change mitigation potential with spatially prioritized allocation and randomized allocation.

Supplementary Text 10 | Scenario simulation process

In our study, we simulated scenarios to evaluate trade-offs between recultivation and reforestation. These simulated scenarios present the outcomes of different land-use allocation approaches and serve to determine how synergies of food production and climate change mitigation could be maximized.

Supplementary Table 8 describes the simulation process, as well as the constraints of parameters. Each simulation is carried out in sequence from Step 1 to Step 3.

Step 1

It starts with determining the amount of abandoned cropland to be allocated in total (A_{total}), and the amount specifically allocated for recultivation ($A_{total\_recultivation}$) and reforestation ($A_{total\_reforestation}$), which are constrained by the total extent of suitable abandoned cropland (i.e., 94Mha for either recultivation or reforestation, 61Mha for recultivation and 83Mha for reforestation).

Step 2

Then, we spatially allocate $A_{total\_recultivation}$ and $A_{total\_reforestation}$ to abandoned cropland pixels suitable for recultivation and reforestation. It should be further noted that each pixel (300-m) is only allocated for one single purpose, either recultivation or reforestation. In other words, the location of the pixel allocated for recultivation (i) should not overlap with the location of the pixel allocated for reforestation (j; i≠j).

Step 3

In the spatial allocation process, we also determine the way of allocation; that is, whether it is allocated in a spatially prioritized way or in a randomized way. Specifically, for simulations without employing a spatial prioritization strategy, pixels are allocated randomly. The food production potential and/or climate change mitigation potential (Step 2) are then determined by the mean outcome of 100-time simulations. For simulations employing a spatially prioritized land allocation strategy, pixels with higher productivity or carbon sequestration rate are allocated first. In this way, the crop productivities and carbon sequestration rates of allocated pixels are higher than those of remaining pixels (not allocated).

Supplementary Table 8 | Flowchart of simulation processes.

**Step**	**Model**	**Constraints**
1	Parameter setting:<br>A_{total}, $A_{total\_recultivation}$, $A_{total\_reforestation}$<br><br>Where:<br>A_{total}, $A_{total\_recultivation}$, $A_{total\_reforestaion}$ represents the total area of abandoned cropland to be allocated, allocated for recultivation and allocated for reforestation.	• $A_{total} = A_{total\_recultivation} + A_{total\_reforestation}$<br>• $0<A_{total}\leq 95$ Mha<br>• $0<A_{total\_recultivation}\leq 61$ Mha<br>• $0<A_{total\_reforestation}\leq 83$ Mha<br><br>Where:<br>95Mha, 61Mha and 83Mha are the extent of abandoned cropland suitable for either recultivation or reforestation, recultivation only, and reforestation only.
2	Allocating pixels of recultivation and reforestation pixel<br>$\begin{cases} Food_i = pixel\_size_i \times Productivity_i \\ Climate_j = pixel\_size_j \times Carbon\_Seq_j \end{cases}$	• $A_{total\_recultivation} = \sum pixel\_size_i$<br>• $A_{total\_reforestation} = \sum pixel\_size_j$

	Where<br>i and j indicate the location of pixel allocated for recultivation and reforestation, respectively; $Food_i$, $pixel\_size_i$, productivity, indicates the food production potential, pixel size (300m*300m), and integrated crop productivity of a recultivatable pixel i; $Climate_j$, $pixel\_size_j$, and $Carbon\_seq_j$ stand for carbon sequestration rates, pixel size (300m*300m), and carbon sequestration rate of a reforestable pixel j. The crop productivity map is derived from Eq. S1, while the carbon sequestration map is derived from Eq. S3-S4.	• i ≠ j
3	Spatial prioritization<br><br>(1) Without employing spatial prioritization<br>i and j are selected randomly. The food production potential and climate change mitigation potential were derived by the mean outcomes of 100-time simulations, using equations in Step 2.<br><br>(2) Employing spatial prioritization<br>i and j are selected by their productivities and carbon sequestration rates, respectively.	• Employing spatial prioritization<br><br>∀ i: $Productivity_i \geq Productivity_{remain}$<br>∀ j: $Carbon\_Seq_j \geq Carbon\_Seq_{remain}$<br><br>Where $Productivity_{remain}$ and $Carbon\_seq_{remain}$ are the productivities and carbon sequestration rates of remaining pixels suitable for recultivation and reforestation, which are not allocated (i.e., not selected) in each simulation.

To better illustrate and compare the simulated outcomes across scenarios, in our analysis we selected and generated four types of hypothetical but representative scenarios **(Supplementary Table 9)**: "maximizing food production" scenario, "maximizing climate change mitigation" scenario, "equal allocation" scenario", and "maximizing combined potential" scenario. For "maximizing combined potential" scenario, whether a pixel is allocated for recultivation or reforestation is determined by its comparative advantage over productivities and carbon sequestration rates.

Below, we describe how these four types of representative scenarios are simulated.

Supplementary Table 9 | Simulation processes of four types of representative scenarios

**Scenario**	**Simulation workflow**
"Maximizing food production"	Step 1 – Step 2 – Step 3<br>If A_{total} >61Mha:<br>$A_{total\_recultivation}$=61Mha<br>$A_{total\_reforestation}$= A_{total} - 61Mha<br>If A_{total} ≤61Mha<br>$A_{total\_recultivation}$= A_{total}

	$A_{total\_reforestation}$= 0
“Maximizing food production”	Step 1 – Step 2 – Step 3<br>If A_{total} >83Mha:<br>$A_{total\_recultivation}$= A_{total} - 83Mha<br>$A_{total\_reforestation}$= 83Mha<br>If A_{total} ≤Mha<br>$A_{total\_recultivation}$= 0<br>$A_{total\_reforestation}$= A_{total}
“Equal allocation”	Step 1 – Step 2 – Step 3<br>$A_{total\_recultivation}$= $A_{total\_reforestation}$
“Maximizing combined potential”	Step 1 – Step 2– Step 3<br>∀ i and j:<br>$Productivity\_rank_i$>$Carbon\_seq_i$<br>$Productivity\_rank_j$>$Carbon\_seq_j$

REFERENCES

1 Clark, M. A., Domingo, N. G. G., Colgan, K., Thakrar, S. K. & Hill, J. D. Global food system emissions could preclude achieving the 1.5° and 2°C climate change targets. *Science* **370**, 705-708 (2020).

2 Fischer, G. *et al.* Global Agro-ecological Zones (GAEZ v4)-Model Documentation. (2021).

3 Hempel, S., Frieler, K., Warszawski, L., Schewe, J. & Piontek, F. A trend-preserving bias correction – the ISI-MIP approach. *Earth System Dynamics* **4**, 219-236, doi:10.5194/esd-4-219-2013 (2013).

4 Chapagain, T. & Good, A. Yield and production gaps in rainfed wheat, barley, and canola in Alberta. *Frontiers in plant science* **6**, 990 (2015).

5 Rosenzweig, C. *et al.* Assessing agricultural risks of climate change in the 21st century in a global gridded crop model intercomparison. *Proceedings of the National Academy of Sciences of the United States of America* **111**, 3268-3273, doi:10.1073/pnas.1222463110 (2014).

6 Xu, S. *et al.* Delayed use of bioenergy crops might threaten climate and food security. *Nature* **609**, 299-306 (2022).

7 Grogan, D., Frolking, S., Wisser, D., Prusevich, A. & Glidden, S. Global gridded crop harvested area, production, yield, and monthly physical area data circa 2015. *Scientific data* **9**, 15, doi:10.1038/s41597-021-01115-2 (2022).

8 Pu, L., Zhang, S., Yang, J., Chang, L. & Bai, S. Spatio-Temporal Dynamics of Maize Potential Yield and Yield Gaps in Northeast China from 1990 to 2015. *Int J Environ Res Public Health* **16**, doi:10.3390/ijerph16071211 (2019).

9 Rattalino Edreira, J. I. *et al.* Spatial frameworks for robust estimation of yield gaps. *Nature Food* **2**, 773-779, doi:10.1038/s43016-021-00365-y (2021).

10 Deng, N. *et al.* Closing yield gaps for rice self-sufficiency in China. *Nat Commun* **10**, 1725, doi:10.1038/s41467-019-09447-9 (2019).

11 Teixeira, E. I., Fischer, G., Van Velthuizen, H., Walter, C. & Ewert, F. Global hot-spots of heat stress on agricultural crops due to climate change. *Agricultural and Forest Meteorology* **170**, 206-215 (2013).

12 Mauser, W. *et al.* Global biomass production potentials exceed expected future demand without the need for cropland expansion. *Nature communications* **6**, 8946 (2015).

13 Chazdon, R. L. *et al.* Fostering natural forest regeneration on former agricultural land through economic and policy interventions. *Environmental Research Letters* **15**, doi:10.1088/1748-9326/ab79e6 (2020).

14 Poorter, L. *et al.* Biomass resilience of Neotropical secondary forests. *Nature* **530**, 211-214, doi:10.1038/nature16512 (2016).

15 Martin, P. A., Newton, A. C. & Bullock, J. M. Carbon pools recover more quickly than plant biodiversity in tropical secondary forests. *Proc Biol Sci* **280**, 20132236, doi:10.1098/rspb.2013.2236 (2013).

16 Crawford, C. L., Yin, H., Radeloff, V. C. & Wilcove, D. S. Rural land abandonment is too ephemeral to provide major benefits for biodiversity and climate. *Science Advances* **8**, eabm8999, doi:doi:10.1126/sciadv.abm8999 (2022).

17 Fradette, O. *et al.* Additional carbon sequestration potential of abandoned agricultural land afforestation in the boreal zone: A modelling approach. *Forest Ecology and Management* **499**, doi:10.1016/j.foreco.2021.119565 (2021).

18 Khorchani, M., Nadal-Romero, E., Lasanta, T. & Tague, C. Carbon sequestration and water yield tradeoffs following restoration of abandoned agricultural lands in Mediterranean mountains. *Environ Res*, 112203, doi:10.1016/j.envres.2021.112203 (2021).

19 Griscom, B. W. *et al.* Natural climate solutions. *Proceedings of the National Academy of Sciences of the United States of America* **114**, 11645-11650, doi:10.1073/pnas.1710465114 (2017).

20 Descals, A. *et al.* High-resolution global map of smallholder and industrial closed-canopy oil palm plantations. *Earth System Science Data* **13**, 1211-1231 (2021).

21 Strassburg, B. B. N. *et al.* Global priority areas for ecosystem restoration. *Nature*, doi:10.1038/s41586-020-2784-9 (2020).

22 Zeng, Y. *et al.* Economic and social constraints on reforestation for climate mitigation in Southeast Asia. *Nature Climate Change* **10**, 842-844, doi:10.1038/s41558-020-0856-3 (2020).

23 Strassburg, B. B. N. *et al.* Strategic approaches to restoring ecosystems can triple conservation gains and halve costs. *Nat Ecol Evol* **3**, 62-70, doi:10.1038/s41559-018-0743-8 (2019).

24 Brancalion, P. H. *et al.* What makes ecosystem restoration expensive? A systematic cost assessment of projects in Brazil. *Biological Conservation* **240**, 108274 (2019).

25 Riahi, K. *et al.* The Shared Socioeconomic Pathways and their energy, land use, and greenhouse gas emissions implications: An overview. *Global Environmental Change* **42**, 153-168, doi:10.1016/j.gloenvcha.2016.05.009 (2017).

26 Fisher, B. *et al.* Implementation and opportunity costs of reducing deforestation and forest degradation in Tanzania. *Nature Climate Change* **1**, 161-164, doi:10.1038/nclimate1119 (2011).

27 Olofsson, P. *et al.* Good practices for estimating area and assessing accuracy of land change. *Remote Sensing of Environment* **148**, 42-57, doi:10.1016/j.rse.2014.02.015 (2014).

28 Yin, H. *et al.* Mapping agricultural land abandonment from spatial and temporal segmentation of Landsat time series. *Remote Sensing of Environment* **210**, 12-24, doi:10.1016/j.rse.2018.02.050 (2018).

29 Olofsson, P., Foody, G. M., Stehman, S. V. & Woodcock, C. E. Making better use of accuracy data in land change studies: Estimating accuracy and area and quantifying uncertainty using stratified estimation. *Remote Sensing of Environment* **129**, 122-131, doi:10.1016/j.rse.2012.10.031 (2013).

30 Verburg, P. H., Ellis, E. C. & Letourneau, A. A global assessment of market accessibility and market influence for global environmental change studies. *Environmental Research Letters* **6**, 034019 (2011).

31 Nelson, A. *et al.* A suite of global accessibility indicators. *Scientific data* **6**, 1-9 (2019).

32 De Groeve, T., Poljansek, K. & Vernaccini, L. Index for risk management-INFORM. *JRC Science for Policy Reports (Brussels: European Commission)* (2015).

33 Reduction, U. N. O. f. D. R. (UNISDR Geneva, Switzerland, 2017).

34 Cook-Patton, S. C. *et al.* Mapping carbon accumulation potential from global natural forest regrowth. *Nature* **585**, 545-550, doi:10.1038/s41586-020-2686-x (2020).
