# Supplementary material for: The neglected role of abandoned cropland in supporting both food security and climate change mitigation
Source: Nat Commun. 2023 Sep 28;14:6083. doi: 10.1038/s41467-023-41837-y (PMC10539403; doi:10.1038/s41467-023-41837-y)
Supplement: Supplementary file 3 — Description of Additional Supplementary Files [file 41467_2023_41837_MOESM3_ESM.pdf]

Description of additional supplementary files

Supplementary Data 1 : Abandoned cropland extent estimated by previous studies.

Supplementary Data 2 : NDC emission reduction targets.

Supplementary Data 3 : Food waste and food loss.

Supplementary Data 4 : Literature review on carbon sequestration rates via active reforestation and natural regeneration.
