# Supplementary figures and images for: The neglected role of abandoned cropland in supporting both food security and climate change mitigation
Source: Nat Commun. 2023 Sep 28;14:6083. doi: 10.1038/s41467-023-41837-y (PMC10539403; doi:10.1038/s41467-023-41837-y)

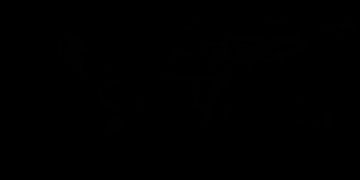

Supplement: Supplementary file 6 — Source Data [file 41467_2023_41837_MOESM6_ESM.zip › source_data_fig1/fig1b-1.tif]

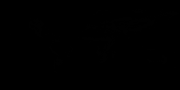

Supplement: Supplementary file 6 — Source Data [file 41467_2023_41837_MOESM6_ESM.zip › source_data_fig1/fig1b-1.tif.ovr]

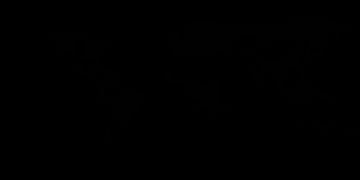

Supplement: Supplementary file 6 — Source Data [file 41467_2023_41837_MOESM6_ESM.zip › source_data_fig1/fig1b-2.tif]

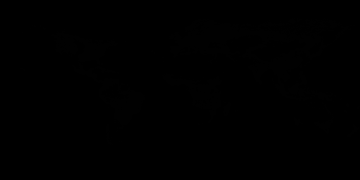

Supplement: Supplementary file 6 — Source Data [file 41467_2023_41837_MOESM6_ESM.zip › source_data_fig1/fig1b-3.tif]
